# Immunogenicity and safety of an adjuvanted inactivated polio vaccine, IPV-Al, compared to standard IPV: A phase 3 observer-blinded, randomised, controlled trial in infants vaccinated at 6, 10, 14 weeks and 9 months of age

**DOI:** 10.1016/j.vaccine.2019.10.064

**Published:** 2020-01-16

**Authors:** Lulu C. Bravo, Josefina C. Carlos, Salvacion R. Gatchalian, May Emmeline B. Montellano, Charissa Fay Corazon B. Tabora, Birgit Thierry-Carstensen, Pernille Nyholm Tingskov, Charlotte Sørensen, Henrik Wachmann, Ananda S. Bandyopadhyay, Pernille Ingemann Nielsen, Mie Vestergaard Kusk

**Affiliations:** a University of the Philippines Manila, Manila, Philippines; b University of the East-Ramon Magsaysay Memorial Medical Center Incorporated, Manila, Philippines; c UP CM University of the Philippines, Manila, Department of Pediatrics. Philippine General Hospital, Philippines; d Mary Chiles General Hospital, Sampaloc, Manila, Philippines; e Research Institute for Tropical Medicine, Muntinlupa City, Metro Manila, Philippines; f Statens Serum Institut, 5 Artillerivej, 2300 Copenhagen S, Denmark; g AJ Vaccines, 5 Artillerivej, 2300 Copenhagen S, Denmark; h Larix A/S, Lyskær 8b, 2730 Herlev, Denmark; i Bill & Melinda Gates Foundation, Seattle, WA, USA

**Keywords:** Affordable IPV, Aluminium hydroxide adjuvant, Booster vaccination, Immunogenicity, Polio, Primary vaccination

## Abstract

**Background:**

A dose-sparing inactivated polio vaccine (IPV-Al), obtained by adsorption of inactivated virus to an aluminium hydroxide adjuvant, can help mitigate global supply and the cost constraints of IPV. The objective of this trial was to demonstrate the non-inferiority of IPV-Al to standard IPV.

**Methods:**

This phase 3, observer-blinded, randomised, controlled trial was conducted at 5 investigational sites in the Philippines. Infants not previously vaccinated with any polio vaccines were randomised to receive three IPV-Al (n = 502) or IPV vaccinations (n = 500) at 6, 10 and 14 weeks of age plus a booster vaccination at 9 months. The primary endpoint was type-specific seroconversion, defined as an antibody titre ≥4-fold higher than the estimated maternal antibody titre and a titre ≥8, one month after the primary vaccination series.

**Results:**

Seroconversion rates following primary vaccination with IPV-Al (483 infants in the per-protocol analysis set) or IPV (478 infants) were: polio type 1, 97.1% versus 99.0%; type 2, 94.2% versus 99.0%; and type 3, 98.3% versus 99.6%. IPV-Al was non-inferior to IPV, as the lower 95% confidence limits of the treatment differences were above the predefined −10%-point limit: type 1, −1.85% (−3.85; −0.05); type 2, −4.75% (−7.28; −2.52); type 3, −1.24 (−2.84; 0.13). The booster effect (geometric mean titre (GMT) post-booster / GMT pre-booster) was: type 1, 63 versus 43; type 2, 54 versus 47; type 3, 112 versus 80. IPV-Al was well tolerated with a safety profile comparable to that of IPV. Serious adverse events were recorded for 29 infants (5.8%, 37 events) in the IPV-Al group compared to 28 (5.6%, 48 events) in the IPV group.

**Conclusion:**

Non-inferiority of IPV-Al to IPV with respect to seroconversion was confirmed and a robust booster response was demonstrated. Both vaccines had a similar safety profile.

ClinicalTrials.gov identifier: NCT03032419.

## 1 Introduction

The world is closer than ever to achieving the aim of polio eradication with more than 99.9% reduction in cases since the time The World Health Assembly launched the Global Polio Eradication Initiative (GPEI) in 1988 [1]. According to the Polio Eradication & Endgame Strategic Plan [2], withdrawal of the oral polio vaccine (OPV), as its use carries a small risk of vaccine-associated paralytic poliomyelitis (VAPP) and circulating vaccine-derived poliovirus (cVDPV) [3], and introduction of inactivated polio vaccine (IPV) are key strategic steps to complete and sustain eradication. The transition to IPV leads to increasing demand for IPV and requires that the constraints in terms of cost and supply availability of IPV are overcome [4]. AJ Vaccines has developed a dose sparing IPV, obtained by adsorption of the inactivated virus to an aluminium hydroxide (Al(OH)_3_) adjuvant, which has enabled the reduction of the amount of antigen by up to ten times compared to the currently used IPV. Promising results of nonclinical studies [5] and clinical trials [6], [7] have demonstrated that reduced-dose vaccines are safe, and their immunogenicity is retained. In a phase 2 observer-blinded, randomised, controlled trial, the immunogenicity and safety of three vaccines with doses of 1/3, 1/5 or 1/10 of the IPV dose were investigated in infants [7]. All three vaccines were non-inferior to IPV with respect to seroconversion rates, with absolute differences in percentage seroconversion for each poliovirus type being greater than the −10% non-inferiority margin. The phase 3 trials were conducted with the adjuvanted IPV with ten-times reduced antigen content (IPV-Al). Advantages of a reduction in antigen content are two-fold: increased availability of IPV and reduced cost, both of major importance for the global eradication programme.

In the present trial, healthy infants from the Philippines received 3 vaccinations of either IPV-Al or standard IPV at 6, 10 and 14 weeks of age, according to the World Health Organization (WHO) expanded programme of immunisation (EPI) schedule [2], plus a booster dose at 9 months. The primary objective was to demonstrate non-inferiority of seroconversion for poliovirus types 1, 2 and 3 for IPV-Al compared to IPV in infants one month after the primary vaccination series. The primary endpoint, type-specific seroconversion, was defined to include infants with both a titre ≥4 times higher than the estimated remaining maternal antibody at 18 weeks and a titre ≥8 at 18 weeks (seroprotection). The endpoint combined measures of the infant’s response to the vaccination (as titres were required to be ≥4 times higher than the estimated levels of maternal antibody) and seroprotection (the established correlate of protection for polio) [8], [9], [10]. Secondary immunogenicity objectives included evaluations of antibody titres one month after the primary vaccination series and the immunogenic responses to the two vaccines following the 9-month booster vaccination. An evaluation of the safety profile of each vaccine was also a secondary objective.

## 2 Methods

### 2.1 Trial design and participants

This was a phase 3, non-inferiority, observer-blinded, randomised (1:1), controlled, multicentre clinical trial with two parallel groups of infants who received three IPV-Al (n = 502) or IPV vaccinations (n = 500) at 6, 10 and 14 weeks of age (primary series) plus a booster vaccination at 9 months of age (a total of 4 vaccinations). Blood samples were taken prior to the vaccination at 6 weeks and at 18 weeks (pre- and post-primary vaccination series) and prior to the vaccination at 9 months and at 10 months (pre- and post-booster vaccination).

The trial was conducted at five investigational sites in Manila or greater Manila, the Philippines. Trial participants were recruited by contacting parents during late pregnancy or when attending the maternity ward. The participants were healthy infants aged 6 weeks on the date of the first vaccination who had not previously been vaccinated with any polio vaccine. Parents/guardians of the infants were informed about the trial and signed consent allowing their child to be included and to receive the trial vaccine. They also granted access to the infant's trial-related medical records. Key exclusion criteria included known exposure to OPV or any wild or vaccine-derived poliovirus in the household within 3 months before inclusion, a low birth weight (<2.5 kg), known or suspected immunodeficiency or family history of congenital or hereditary immunodeficiency, severe uncontrolled chronic disease, and known or suspected allergy to the vaccine constituents.

The trial protocol was approved by the relevant ethics committees and competent authorities prior to trial commencement and the trial was conducted according to the principles of good clinical practice [11] and the Declaration of Helsinki [12]. The trial is registered with ClinicalTrials.gov (NCT03032419).

### 2.2 Trial vaccines

The investigational and comparator vaccines were both manufactured by Statens Serum Institut (now manufactured by AJ Vaccines) (Copenhagen S, Denmark)^1^ and contained inactivated poliovirus types 1 (Brunhilde), 2 (MEF-1) and 3 (Saukett). The comparator vaccine was a licenced non-adjuvanted standard IPV vaccine containing 40 D-antigen units (DU) of poliovirus type 1, 8 DU of type 2 and 32 DU of type 3, and appearing as a clear solution for injection. The investigational IPV-Al vaccine was also a trivalent IPV vaccine containing approximately one tenth of the amount of each antigen in the IPV vaccine, adjuvanted to aluminium hydroxide (0.5 mg aluminium), appearing as an opaque suspension for injection.

One vaccine dose of 0.5 mL IPV-Al or standard IPV was administered intramuscularly perpendicular to the skin in the anterolateral aspect of the thigh using a sterile disposable syringe fitted with a 23-gauge, 25 mm needle. In addition to the trial vaccinations, the infants received concomitant childhood vaccinations during the trial period. The trial vaccines were administered in the right thigh, whereas the other injectable childhood vaccines were administered in the left thigh.

### 2.3 Randomisation and blinding

A randomisation list was generated by an unblinded statistician using SAS software, version 9.4 (SAS Institute, Cary, NC, USA), who did not otherwise participate in the trial. The list was uploaded into the electronic case report form so that the site could automatically randomise the trial participants. Each randomised infant was given the lowest available subject number.

As the two vaccine formulations were visually distinguishable, the observer-blinded nature of the trial meant that only prespecified unblinded site staff and the trial monitor had access to the trial vaccines and dispensing logs during the trial. Only unblinded site personnel and parents were present during administration of the vaccines, and the investigators and other individuals associated with the trial remained blinded.

### 2.4 Immunogenicity endpoints

The primary endpoint was the proportion of infants with type-specific seroconversion one month after the primary vaccination series, defined as (1) a post-vaccination antibody titre ≥4-fold higher than the estimated maternal antibody titre, based on the pre-vaccination titre declining by a half-life of 28 days, and (2) a post-vaccination titre ≥8. The established correlate of protection against polio is a neutralising antibody titre of ≥8 for the poliovirus types 1, 2 and 3 [8], [9], [10].

The two components of the primary endpoint were also included separately as secondary endpoints. Thus, secondary immunogenicity-related endpoints included the proportion of infants with seroprotection (antibody titre ≥8) against poliovirus types 1, 2 and 3, respectively, one month after the primary vaccination series and before and one month after the booster vaccination at 9 months. Geometric mean titres (GMTs) and median antibody titres were also measured. The proportion of infants with post-vaccination titres ≥4-fold higher than the estimated maternal antibody titre was evaluated for both vaccines. The booster effects (GMT post-booster/GMT pre-booster = GMTR) were assessed, as well as the booster effect ratios (GMTR IPV-Al/GMTR IPV) from the 9 months (pre-booster) and 10 months (post-booster) antibody titre measurements for the three poliovirus types. Antibody persistence, measured as the decline in titres from one month after the primary vaccination series to the time of the booster vaccination, was compared between the two vaccines.

A subgroup analysis of the proportion of infants with seroconversion one month after the primary vaccination series, as defined by the primary endpoint, was performed in infants with seroprotection (titre ≥8) and without seroprotection (titre <8) at baseline (week 6). The proportion with seroprotection (titre ≥8) one month after the primary vaccination series as well as one month after the booster vaccination at 9 months was also analysed.

A validated Vero cell assay was used for the antibody titre determinations and has been described previously [6], [13].

### 2.5 Safety endpoints

The infants remained at the investigational site for 30 min after each vaccination under observation by the trial staff in case of adverse events (AEs). Thereafter, the parents/guardians were given a diary, ruler and thermometer and, on the vaccination day and subsequent 2 days, were to measure and report in the diary details of any injection site redness (erythema)/swelling (measured in mm) at the injection site of the study vaccines, axillary temperature, persistent crying, irritability, drowsiness, loss of appetite and vomiting (solicited AEs), as well as any other AEs. Information on any concomitant medication or other vaccines administered between the trial visits was also reported. During days 3–6 after each vaccination, the parents/guardians were to complete details in the diary only if they detected an AE, or if the infant received medication or other vaccines between the trial visits. The site investigator interviewed the parents/guardians at the subsequent trial visit, assessed all AEs according to their causality, intensity, outcome and seriousness, and transferred the information to the electronic case report form. Safety endpoints were solicited injection site events, solicited systemic AEs and all other AEs following the vaccinations.

### 2.6 Statistical analyses

Randomised controlled clinical vaccine trials tend to use a non-inferiority margin of either 5% or 10% [14]; the choice of margin is dependent on different factors. For the primary immunogenicity endpoint, a non-inferiority margin of 10% was applied. This margin was justified by the strict primary endpoint requiring both seroconversion (a post-vaccination titre ≥4-fold higher than the estimated maternal antibody titre) and seroprotection (a post-vaccination titre ≥8), which is stricter than seroprotection only, together with the fact that the risk of poliomyelitis infection has decreased significantly in recent years. For the secondary immunogenicity endpoint, seroprotection, a narrower non-inferiority margin of 5% was chosen.

The sample size calculation was based on seroconversion rates (ranging from 94.6% to 100%) demonstrated in the phase 2 dose-investigation clinical trial [7]. If the true seroconversion for IPV-Al was lower than for IPV, e.g. by 5 percentage points for all 3 poliovirus types, then 350 participants per treatment group was considered sufficient to achieve a power of >80%. Based on these considerations, a non-inferiority margin of 10% for the primary immunogenicity endpoint analysis, and allowing for possible exclusion of up to 15% of infants (due to withdrawals or major protocol deviations), as well as advice from the European Medicines Agency, a sample size of 500 infants per group was to be included.

The primary immunogenicity analyses were based on the per-protocol (PP) analysis set (defined as all participants in the full analysis set (FAS) who had no major protocol deviations). Sensitivity analyses of the primary endpoint were performed using the FAS (defined as all randomised and vaccinated infants with a valid primary endpoint for at least 1 poliovirus type). In order to qualify for the primary analysis, the availability of each of the components of the primary endpoint (a type-specific post-vaccination antibody titre ≥4-fold higher than the estimated maternal antibody titre as well as a type-specific post-vaccination titre ≥8) was required. For a given poliovirus type, if either baseline or post-vaccination titres were missing, the seroconversion result was assigned a missing value. The estimated maternal antibody titre was calculated as:

$$\text{Titre}\left(\text{t}\right)=\text{titre}\left(\text{baseline}\right)\times \text{exp}\left(-\left(\text{ln}\left(2\right)/\text{t}1/2\right)\times \text{t}\right)$$

where t was the time in days since baseline and t½ was the expected half-life of maternal antibodies of 28 days [15].

Non-inferiority of IPV-Al to IPV was demonstrated only if non-inferiority of the seroconversion rates was achieved for each of the 3 poliovirus types 1, 2 and 3; hence, no multiplicity adjustment was needed. Each of the primary immunogenicity analyses was evaluated by calculating the rate difference (P_i, IPV-Al_ – P_i, IPV_) with a two-sided 95% confidence interval (CI) based on observed cases, i.e. disregarding missing values. The 95% CI was calculated as an approximative Newcombe-Wilson interval [16], and non-inferiority could be claimed if the lower 95% confidence limit was greater than the −10% non-inferiority margin. This comparison corresponded to a one-sided test at a significance level of 2.5%.

The non-inferiority testing used for the primary endpoint in the PP analysis set was repeated for the seroprotection endpoint one month after the primary vaccination series, using a 5% margin. The rate differences and confidence limits for the secondary immunogenicity endpoints were derived in the same way as for the primary endpoint. Geometric mean and median titres for poliovirus types 1, 2 and 3 as well as safety endpoints were summarised using descriptive statistics. SAS software, version 9.4, was used for the analyses.

## 3 Results

### 3.1 Population characteristics

**Fig. 1:**
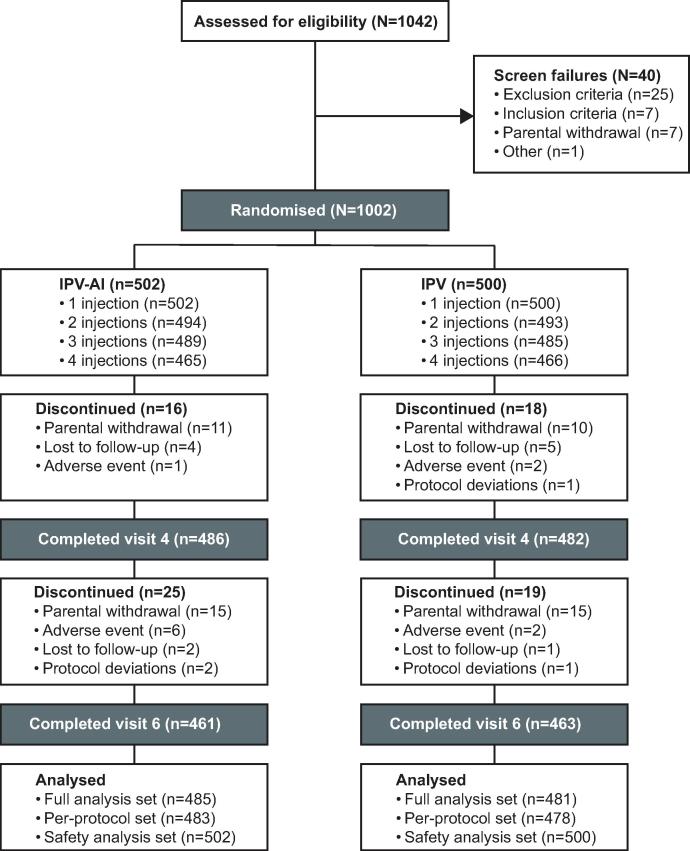
Trial profile. Visit 4 was one month after the primary vaccination series at 14 weeks. Visit 6 was one month after the booster vaccination at 9 months. The full analysis set (FAS) was defined as all randomised and vaccinated infants with a valid primary endpoint for at least one poliovirus type. The per-protocol analysis set was defined as all infants in the FAS who had no major deviations from the protocol. The safety analysis set was defined as all randomised infants who received at least one treatment dose. N = number of infants in the group.

The trial was conducted between 6 February 2017 (the first trial visit) and 12 March 2018 (the last trial visit). In the trial, 1042 potential participants were screened and assessed for eligibility, of which 40 were screen failures (Fig. 1). The enrolled participants (N = 1002) were randomised to receive either the IPV-Al vaccine (n = 502) or the comparator IPV (n = 500) vaccine. Overall, 968 infants (96.6%) completed the trial visit one month after the primary vaccination series and 924 (92.2%) infants completed the last trial visit one month after the booster vaccination (Fig. 1). Two infants in the IPV-Al group and 3 in the IPV group were excluded from the PP analysis set due to major protocol deviations.

Both vaccination groups were comparable in terms of demography and baseline characteristics (Table 1).

**Table 1:** Baseline characteristics of the trial population.

Characteristic	IPV-Al N = 502	IPV N = 500
Sex, n (%)		
Male	255 (50.8)	255 (51.0)
Female	247 (49.2)	245 (49.0)
Race Asian, n (%)	502 (100)	500 (100)
Age, days	45.06 (4.3)	45.06 (4.6)
Birth weight, kg	3.05 (0.36)	3.05 (0.35)

Data are n (%) or mean (SD) for participants in the safety analysis set.

N = number of infants, SD = standard deviation.

### 3.2 Immunogenicity results

The seroconversion (primary endpoint) rates for poliovirus types 1, 2 and 3 following primary immunisation are presented in Table 2. Seroconversion rates after vaccination with three doses of IPV-Al compared to IPV were: type 1: 97.1% versus 99.0%; type 2: 94.2% versus 99.0%; and type 3: 98.3% versus 99.6%. Non-inferiority of IPV-Al to IPV with respect to the primary endpoint of seroconversion was demonstrated for each of the 3 poliovirus types, as indicated by the lower limit of the two-sided 95% CIs of the rate differences being above the predefined limit of −10%-points (Table 2).

**Table 2:** Non-inferiority analysis of seroconversion (primary endpoint) and secondary endpoint seroprotection (titre ≥ 8) rates one month after the primary vaccination series.

Poliovirus type	IPV-Al N = 483 % (95% CI)	IPV N = 478 % (95% CI)	Treatment difference (95% CI)
**Seroconversion**			

Type 1	97.1 (95.2 to 98.4)	99.0 (97.6 to 99.7)	−1.85 (-3.85 to −0.05)
Type 2	94.2 (91.7 to 96.1)	99.0 (97.6 to 99.7)	−4.75 (-7.28 to −2.52)
Type 3	98.3 (96.8 to 99.3)	99.6 (98.5 to 99.9)	−1.24 (-2.84 to 0.13)

**Seroprotection**			

Type 1	97.9 (96.2 to 99.0)	99.6 (98.5 to 99.9)	−1.65 (-3.38 to −0.21)
Type 2	100 (99.2 to 100)	99.6 (98.5 to 99.9)	0.42 (-0.43 to 1.51)
Type 3	99.0 (97.6 to 99.7)	99.8 (98.8 to 100)	−0.83 (-2.20 to 0.31)

Data are presented for the per-protocol analysis set. The primary endpoint was defined as (1) a type-specific post-vaccination titre ≥4-fold higher than the estimated maternal antibody titre, based on the pre-vaccination titre declining by a half-life of 28 days, and (2) a type-specific post-vaccination titre ≥8.

CI = confidence interval, N = number of infants in trial group.

In a sensitivity analysis of the primary seroconversion endpoint in the FAS, no major difference between the PP and the FAS population results was observed and non-inferiority of IPV-Al to IPV was also demonstrated in the FAS (treatment difference of −1.85%, −4.73% and −1.23% for type 1, 2 and 3 poliovirus types, respectively).

The two components of the primary endpoint were also analysed separately in the PP population. For IPV-Al compared with IPV, the rates for titres ≥4-fold the estimated maternal antibody titres were: type 1: 99.2% versus 99.2%; type 2: 94.2% versus 99.0%; and type 3: 99.2% versus 99.6%. In addition, between 97.9 and 100% of infants in the IPV-Al group and between 99.6 and 99.8% of those in the IPV group were seroprotected (antibody titres ≥8) after the primary vaccination series (Table 2), and non-inferiority (5%-margin) of the seroprotection of IPV-Al to IPV was confirmed across poliovirus types.

The baseline GMTs at week 6 were similar in both vaccine groups (Table 3). The post-primary vaccination GMTs were high for all poliovirus types, regardless of treatment group, and were higher with IPV than with IPV-Al. A decline in GMTs from the post-primary vaccination series to the booster vaccination at 9 months was observed for all poliovirus types regardless of vaccine used. The decline appeared to be similar in both groups (supplementary figure), although the GMTs remained higher with IPV than IPV-Al.

**Table 3:** Summary of seroprotection rates and serum neutralising antibody titres for poliovirus types 1, 2 and 3.

Poliovirus type and endpoint	Baseline (age 6 weeks)		Post-primary vaccination (age 18 weeks)		Pre-booster vaccination (age 9 months)		Post-booster vaccination (age 10 months)	
	IPV-Al n = 483	IPV n = 478	IPV-Al n = 483	IPV n = 478	IPV-Al n = 449	IPV n = 449	IPV-Al n = 441	IPV n = 442
**Type 1**								

Seroprotection (titre ≥ 8), %	60.5	60.7	97.9	99.6	88.0	100.0	99.8	100.0
GMT (95% CI)	12.2 (10.6 to 14.1)	12.2 (10.6 to 14.1)	739.8 (635 to 862)	3837 (3493 to 4216)	132.3 (109 to 160)	746.2 (666 to 836)	8394 (7122 to 9894)	31,558 (28798 to 34582)
Median titre	11.3	11.3	1024	4096	181	724	11,585	32,768

**Type 2**								

Seroprotection (titre ≥ 8), %	88.0	90.4	100.0	99.6	99.8	99.8	100.0	100.0
GMT (95% CI)	50.0 (43.4 to 57.6)	49.8 (43.5 to 57.1)	1272 (1135 to 1425)	3611 (3279 to 3976)	351.8 (314 to 395)	748.5 (679 to 825)	18,933 (17033 to 21045)	35,164 (32239 to 38355)
Median titre	64.0	45.3	1448	4096	362	724	16,384	32,768

**Type 3**								

Seroprotection (titre ≥ 8), %	55.3	57.5	99.0	99.8	93.5	99.8	100.0	100.0
GMT (95% CI)	9.9 (8.7 to 11.2)	11.6 (10.1 to 13.3)	1110 (974 to 1265)	4590 (4155 to 5071)	142.9 (121 to 169)	633.6 (559 to 718)	15,691 (13767 to 17883)	49,964 (45533 to 54826)
Median titre	8.0	11.3	1448	4096	181	724	16,384	46,341

Data are presented for the per-protocol analysis set. CI = confidence interval, GMT = geometric mean titre, n = number of infants in trial group.

There was a sharp increase in antibody titres following the booster vaccination with both vaccines (Table 3). The post-booster GMTs were higher with IPV than IPV-Al for all poliovirus types. Regardless of vaccine, the GMTs were well in excess of the seroprotection threshold. The post-booster seroprotection rates for both vaccination groups were high, ranging from 99.8 to 100% of infants across poliovirus types (Table 3); only one infant (in the IPV-Al group) did not have seroprotection after the booster vaccination.

The booster effect (GMT post-booster/GMT pre-booster = GMTR) for the IPV-Al group compared with the IPV group was: 63 (95% CI 54; 73) vs 43 (37; 50) for type 1, 54 (48; 61) vs 47 (42; 53) for type 2 and 112 (95; 131) vs 80 (68; 93) for type 3. The booster effect ratios (GMTR IPV-Al/GMTR IPV) were 1.46 (1.18; 1.80) for type 1, 1.15 (0.97; 1.36) for type 2 and 1.40 (1.12; 1.75) for type 3.

**Fig. 2:**
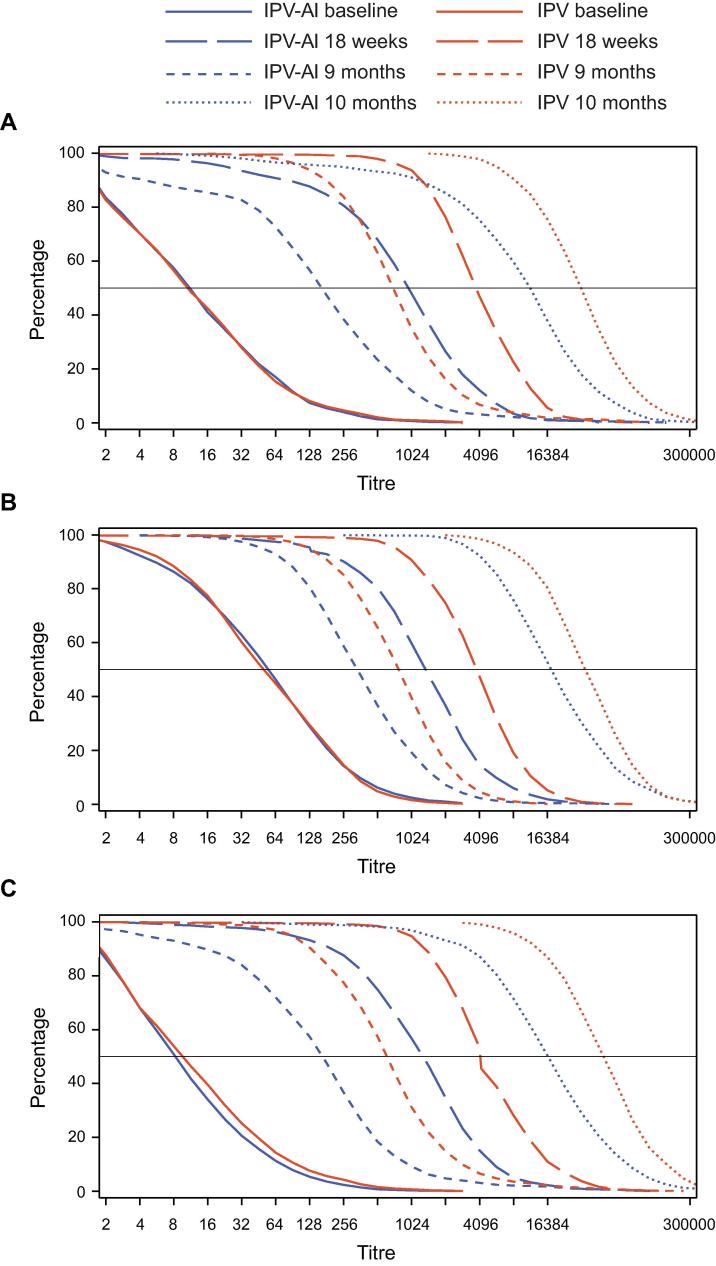
Reverse cumulative antibody titre distribution curves at baseline (6 weeks), post-primary vaccination (18 weeks), pre-booster (9 months) and post-booster vaccination (10 months) for poliovirus type 1 (A), type 2 (B) and type 3 (C).

Reverse cumulative titre distribution curves (Fig. 2) illustrate the changes in antibody titre distributions before and after the primary and booster vaccinations. For each poliovirus type, the baseline curves are almost identical in the two groups. The post-primary vaccination curves illustrate that both vaccines were immunogenic with similar curve shapes for the two vaccines across poliovirus types. The pre-booster vaccination curves illustrate the decline in antibody levels from post- 3rd primary vaccination to pre-booster vaccination. The post-booster vaccination curves illustrate an increase in antibody titres for all poliovirus types in both vaccination groups. The shape of the post-primary and post-booster vaccination curves reflects the wider distribution of titres for type 1 and 3 serotypes than for type 2 in both IPV-Al and IPV vaccinated infants.

In a subgroup analysis, more than 93.4% of infants in both treatment groups obtained seroconversion (primary endpoint) across all 3 poliovirus types following the primary vaccination series, irrespective of their seroprotection status at baseline (week 6). Similarly, more than 94.8% of infants in both treatment groups were seroprotected after the primary vaccination series across all 3 poliovirus types, irrespective of their seroprotection status at baseline, and all except for one infant in the IPV-Al group (titre of 5.7 for poliovirus type 1) were seroprotected after the booster vaccination.

### 3.3 Safety results

In total, 489 of 502 infants (97.4%) in the IPV-Al group and 492 of 500 infants (98.4%) in the IPV group had at least one AE reported by their parent/guardian over the course of the trial. The proportion of serious AEs was similar between groups: 29 infants (5.8%) in the IPV-Al group had 37 serious AEs recorded and 28 (5.6%) in the IPV group had 48 serious AEs recorded. All serious AEs were assessed as not related to the trial vaccine, except for one case of febrile seizure (IPV group) that was assessed as possibly related to treatment.

Five cases had a fatal outcome during the trial: two in the IPV-Al group due to pneumonia and sudden infant death syndrome and three in the IPV group due to gastroenteritis, parasitic gastroenteritis, pneumonia and febrile convulsion; hypovolemic shock and malnutrition; and pneumonia. None of the deaths were assessed as being related to the trial vaccines.

Overall, the proportion of infants with at least one injection site reaction was similar between the IPV-Al (46.2%) and IPV (42.2%) treatment groups (Table 4). All injection site reactions were assessed as either mild or moderate in intensity. Almost all events were either erythema (40.4% of infants in the IPV-Al group and 38.4% in the IPV group) or swelling (34.9% and 29.6%, respectively). Most of the cases of erythema (94%) and swelling (85%) were <25 mm in diameter; less than 2% had a diameter ≥50 mm. The proportion of infants with at least one systemic AE was also similar between IPV-Al (97.2%) and IPV (98.0%) vaccines. The four most common systemic AEs were pyrexia, upper respiratory tract infection, irritability and somnolence. Almost all events (98%) were of mild intensity; 0.3% were severe, and most (62.9%) were considered related to treatment.

**Table 4:** Summary of adverse injection site reactions and adverse events with a frequency of at least 5% of infants in any group.

Adverse event type	IPV-Al N = 502 n (%) e	IPV N = 500 n (%) e
**Mild adverse injection site reactions**	230 (45.8) 848	208 (41.6) 715
Injection site erythema	202 (40.2) 488	191 (38.2) 407
Injection site swelling	171 (34.1) 356	144 (28.8) 304
Injection site pain	3 (0.6) 3	2 (0.4) 2
Injection site bruising	1 (0.2) 1	1 (0.2) 1
Injection site dermatitis	–	1 (0.2) 1

**Moderate adverse injection site reactions**	12 (2.4) 19	15 (3.0) 18
Injection site swelling	11 (2.2) 15	12 (2.4) 12
Injection site erythema	4 (0.8) 4	4 (0.8) 5
Injection site pain	–	1 (0.2) 1

**AEs with frequency ≥ 5%**	488 (97.2) 3139	490 (98.0) 3322
Pyrexia	425 (84.7) 838	448 (89.6) 980
URTI	284 (56.6) 503	279 (55.8) 469
Irritability	232 (46.2) 427	250 (50.0) 514
Somnolence	203 (40.4) 381	220 (44.0) 419
Rhinitis	99 (19.7) 127	67 (13.4) 84
Decreased appetite	77 (15.3) 119	88 (17.6)115
Pneumonia	74 (14.7) 94	72 (14.4) 85
Systemic viral infection	62 (12.4) 77	70 (14.0) 84
Crying	38 (7.6) 51	58 (11.6) 88
Gastroenteritis	51 (10.2) 57	43 (8.6) 49
Nasopharyngitis	51 (10.2) 55	34 (6.8) 35
Vomiting	43 (8.6) 52	36 (7.2) 47
Viral rash	27 (5.4) 28	23 (4.6) 24
Bronchitis	18 (3.6) 18	25 (5.0) 26

Data are for infants in the safety analysis set. AEs are presented by MedDRA (version 19.1) preferred term.

AE = adverse event, e = number of events, MedDRA = Medical Dictionary for Regulatory Activities, N = number of infants in group, n (%) = number (percentage) of infants with AE, URTI = upper respiratory tract infection.

## 4 Discussion

In the present phase 3 trial, the IPV-Al vaccine demonstrated high post-primary vaccination (WHO EPI schedule) seroconversion rates ranging from 94.2 to 98.3% across poliovirus types, comparable to those of IPV, which ranged from 99.0 to 99.6%. Non-inferiority of IPV-Al to IPV was confirmed with respect to the primary endpoint of seroconversion, using a non-inferiority margin of 10%, for each poliovirus type in the trial population of healthy infants from the Philippines. Following the primary vaccination series, seroprotection levels ranged from 97.9% to 100% for IPV-Al and from 99.6% to 99.8% for IPV, and non-inferiority of IPV-Al to IPV was confirmed. One month after the booster vaccination at 9 months, a clear immune response was demonstrated for both vaccines, with increases in both seroprotection rates and GMT levels. Antibody titre declines during the 6-month period from completed priming until the booster vaccination appeared to be similar in the IPV-Al and IPV groups. Overall, the results of the present trial support the applicability of the dose-sparing IPV-Al vaccine to help secure a stable supply of IPV for prevention of poliomyelitis.

The results of the present trial confirm those previously observed with the IPV-Al. In a phase 2 randomised, controlled trial [7], the IPV-Al vaccine was also non-inferior to IPV as regards seroconversion rates according to the same definition. In both trials, the post-primary vaccination GMTs for the three poliovirus types were lower in the IPV-Al group than the IPV group, most likely due to the lower antigen content of the IPV-Al vaccine. Regardless of vaccine, the GMTs were well in excess of the seroprotection threshold. After primary vaccination, the large majority of infants seroconverted (IPV-Al ≥94.2%; IPV ≥99.0% across poliovirus types) and were seroprotected (antibody titres ≥8) (IPV-Al ≥97.9%; IPV ≥99.6% across poliovirus types). Thus, in the present trial, it is considered unlikely that the lower GMTs in the IPV-Al group are clinically important. Irrespective of seroprotection status at baseline, high seroconversion and seroprotection rates were observed following primary vaccination. The booster vaccination administered approximately 6 months after completion of the primary vaccination series induced a clear immune response. Following the booster vaccination, there were no differences between the two vaccines in seroprotection rates across poliovirus types (99.8%-100% for IPV-Al and 100% for IPV), and seroprotection was obtained for all but one infant in the IPV-Al group. The pronounced booster effect indicates that although antibody titres had declined 6 months after the primary vaccination series, immunological priming ensured continued protection through an efficient booster response. Lasting immunity depends on adequate priming i.e. establishment of immune memory (generation of B memory cells). Immunological priming after vaccination with IPV-Al was demonstrated by robust booster effects and increase in post-booster GMTs compared to post-primary GMTs. The strong booster response after vaccination with IPV-Al indicates that immune memory has been established during the primary vaccination. Consequently, should titre levels fall below the protective threshold over time, following antigenic re-stimulation (from vaccine or poliovirus) antibody concentrations are expected to return to above the seroprotective threshold in virtually all of the population vaccinated with IPV-Al. A booster response was previously observed in a phase 1/2 trial, in which three reduced-dose vaccines, including the 1/10 IPV-Al used in the present trial, were administered as a booster dose to adolescents previously vaccinated with IPV at 3, 5 and 12 months and at 5 years [6]. Thus, IPV-Al does induce a booster effect in both IPV and IPV-Al primed individuals. The comparator used in this trial, IPV, was the same as that used in other trials investigating IPV-Al [17], [18].

In areas where poliovirus is endemic, early immunisation of infants is important and, according to the WHO EPI schedule [2], primary vaccination of infants at 6, 10 and 14 weeks of age is recommended, as used in the present trial. New-born infants are protected from polio by maternal antibodies transferred from the mother during the second trimester of pregnancy [19]. This passive immunity declines in a time-dependent manner as the maternal antibodies decay. Maternal antibodies have been shown to interfere with the infant serologic response to IPV vaccination [20]. Vaccinations given at 6, 10 and 14 weeks, the EPI schedule, is thus considered a more challenging schedule than the schedule at 2, 4 and 6 months due to the presence of more maternal antibodies.

Previous trials have also investigated the use of reduced doses of IPV compared to standard IPV, with the ultimate aim of obtaining a more affordable vaccine for infant vaccination programmes, especially in countries where low cost vaccines are urgently needed. Dose sparing by means of intradermal administration has demonstrated varying results in the EPI schedule [18], [21], [22], [23]. We have shown that dose sparing by means of intramuscular administration of a low-dose aluminium hydroxide adjuvanted IPV can be effective even when using the EPI schedule. In light of the insufficient global IPV supply to meet demand, new dose-sparing strategies have the potential not only to ameliorate supply availability issues but also to reduce immunisation costs [24].

The GMT levels following IPV-Al vaccination were lower than with IPV. Since the rate of decline in antibodies was similar for IPV-Al and IPV, the GMTs were also lower prior to the booster vaccination and there was no adjustment for pre-booster titre levels, which could be considered a limitation of the trial. A strength of the trial is the choice of primary endpoint, which combines measures of the infant’s own response to the vaccination (post-vaccination titre ≥4-fold higher than the estimated maternal antibody titre) and the immunological correlate for protection (post-vaccination titre ≥8), i.e. only infants that are both vaccine responders and have a titre above the limit of protection are considered to meet the primary endpoint. This is a stricter definition than the usual practice of defining seroconversion and seroprotection as separate endpoints.

The IPV-Al vaccine was well tolerated, with a safety profile comparable to that of standard IPV with respect to adverse events, including injection site reactions, as seen in previous trials with IPV-Al in adolescents and infants [6], [7]. Previous clinical trials and post-marketing experience with IPV and aluminium hydroxide adjuvanted IPV in different combinations have not revealed any safety concerns [17], [25], [26], [27], [28], [29]. Although there were five infant deaths in the trial, two in the IPV-Al group and three in the IPV group, none were assessed as being related to the trial vaccines. The rate of deaths in the trial was not higher than expected based on the infant mortality rate in the Philippines (in 2015, the mortality rates for age 0 to 1 year was 22 deaths per 1000 infants per year) [30].

## 5 Conclusions

The IPV-Al vaccine demonstrated high seroconversion and seroprotection rates one month after completion of the EPI vaccination schedule comparable to those of standard IPV. Non-inferiority of seroconversion (non-inferiority margin of 10%) and seroprotection rates (non-inferiority margin of 5%) was confirmed for all poliovirus types. IPV-Al induced a robust booster response in 9-month-old infants, and with seroprotection rates comparable to those achieved with IPV one month after the booster vaccination. While the post-vaccination GMTs were higher with IPV than with IPV-Al, they were well in excess of the seroprotection threshold for both vaccines. IPV-Al was well tolerated, with a safety profile comparable to that of standard IPV. IPV-Al has the promise to be a key tool in the final phases of the polio eradication endgame and is probably the most advanced among all the research and development initiatives targeted to mitigate the cost and supply constraints related to the use of inactivated polio vaccines [31]. The novel clinical evidence reported here supports the applicability of IPV-Al vaccine to help secure a stable supply of IPV and use of the vaccine in the target populations in the near future.

## Declaration of Competing Interest

The authors declare the following financial interests/personal relationships which may be considered as potential competing interests: [Birgit Thierry-Carstensen and Pernille Nyholm Tingskov are employees of Statens Serum Institut, a governmental non-profit research organization, and Charlotte Sørensen, Pernille Ingemann Nielsen and Mie Vestergaard Kusk are employees of AJ Vaccines A/S. Both Statens Serum Institut and AJ Vaccines A/S were involved in developing the vaccine at the time the trial was conducted. May Emmeline B. Montellano received research grants from Sanofi Pasteur and SK Chemicals. Ananda S. Bandyopadhyay is a full-time employee at the Bill & Melinda Gates Foundation].

## Footnotes

1 The development of IPV-Al was initiated by Statens Serum Institut (SSI) and was transferred to AJ Vaccines upon its establishment in January 2017.
