# Supplementary material for: Immunogenicity and safety of an adjuvanted inactivated polio vaccine, IPV-Al, compared to standard IPV: A phase 3 observer-blinded, randomised, controlled trial in infants vaccinated at 6, 10, 14 weeks and 9 months of age
Source: Vaccine. 2020 Jan 16;38(3):530–8. doi: 10.1016/j.vaccine.2019.10.064 (PMC6983932; doi:10.1016/j.vaccine.2019.10.064)

# ONLINE SUPPLEMENTARY MATERIAL

Immunogenicity and safety of a low-dose adjuvanted inactivated polio vaccine, IPV-Al, compared to standard IPV: a phase 3 observer-blinded, randomised, controlled trial in infants vaccinated at 6, 10, 14 weeks and 9 months of age

Lulu C. Bravo^a^, Josefina C. Carlos^b^, Salvacion R. Gatchalian^c^, May Emmeline B. Montellano^d^, Charissa Fay Corazon B. Tabora^e^, Birgit Thierry-Carstensen^f^, Pernille Nyholm Tingskov^g^, Charlotte Sørensen^h^, Henrik Wachmann^i^, Ananda S. Bandyopadhyay^j^, Pernille Ingemann Nielsen^k^, Mie Vestergaard Kusk^l^

*^a^University of the Philippines Manila, Manila. Philippines. lulubravo@ymail.com*

*^b^University of the East-Ramon Magsaysay Memorial Medical Center Incorporated, Manila, Philippines. jccarlosmd@gmail.com*

*^c^UP CM University of the Philippines, Manila, Department of Pediatrics. Philippine General Hospital. sallymd77@yahoo.com*

*^d^Mary Chiles General Hospital, Sampaloc, Manila, Philippines. maymd16@gmail.com*

*^e^Research Institute for Tropical Medicine, Muntinlupa City, Metro Manila, Philippines. cbtabora@yahoo.com*

*^f^Statens Serum Institut, 5 Artillerivej, 2300 Copenhagen S, Denmark. BTC@ssi.dk*

*^g^Statens Serum Institut, 5 Artillerivej, 2300 Copenhagen S, Denmark. PNT@ssi.dk*

*^h^AJ Vaccines, 5 Artillerivej, 2300 Copenhagen S, Denmark. CHS@ajvaccines.com*

*^i^Larix A/S, Lyskær 8b, 2730 Herlev, Denmark. HEW@larixcro.com*

*^j^Bill & Melinda Gates Foundation, Seattle, WA, USA. Ananda.bandyopadhyay@gatesfoundation.org*

*^k^AJ Vaccines, 5 Artillerivej, 2300 Copenhagen S, Denmark. PXN@ajvaccines.com*

*^l^AJ Vaccines, 5 Artillerivej, 2300 Copenhagen S, Denmark. MHK@ajvaccines.com*

**Corresponding author**: Mie Vestergaard Kusk

Email address: MHK@ajvaccines.com

# Figure S1: Geometric mean titres (GMTs) in infants vaccinated with IPV-Al and IPV at baseline (2 months), post-primary vaccination (18 weeks), pre-booster (9 months) and post-booster (10 months) for poliovirus type 1 (A), type 2 (B) and type 3 (C). GMTs are shown as log2 values.


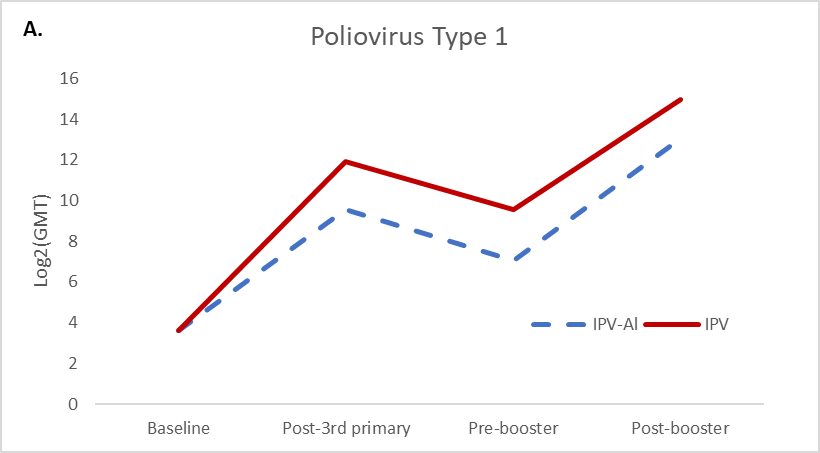


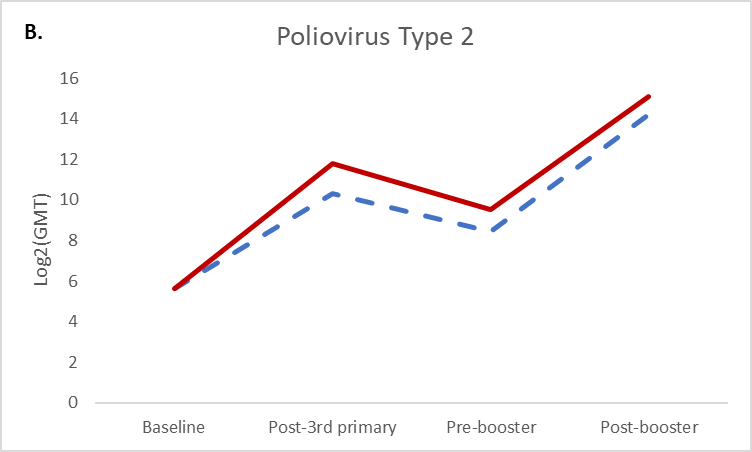


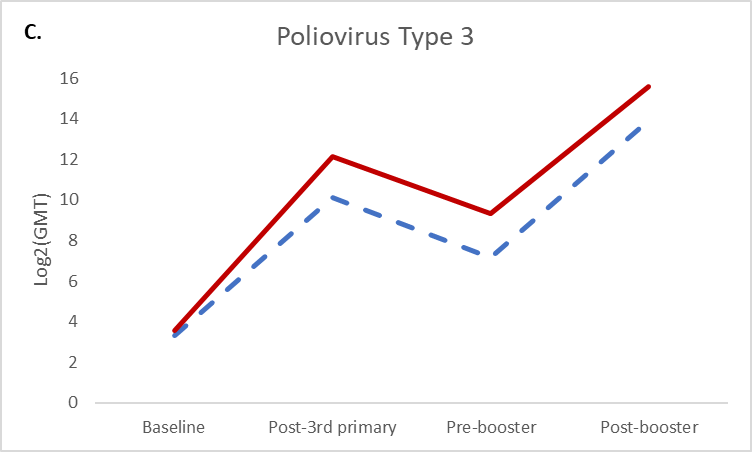

Supplement: Supplementary data 1 [file mmc1.doc]
